# Disparities in Cisplatin-Induced Cytotoxicity—A Meta-Analysis of Selected Cancer Cell Lines

**DOI:** 10.3390/molecules28155761

**Published:** 2023-07-30

**Authors:** Małgorzata Ćwiklińska-Jurkowska, Małgorzata Wiese-Szadkowska, Sabina Janciauskiene, Renata Paprocka

**Affiliations:** 1 Department of Biostatistics and Biomedical Systems Theory, Faculty of Pharmacy, Ludwik Rydygier Collegium Medicum, Nicolaus Copernicus University in Toruń, Jagiellońska Str. 15, 87-067 Bydgoszcz, Poland; mjurkowska@cm.umk.pl; 2 Department of Immunology, Faculty of Pharmacy, Ludwik Rydygier Collegium Medicum, Nicolaus Copernicus University in Toruń, M. Curie-Sklodowska Str. 9, 85-094 Bydgoszcz, Poland; 3 Department of Respiratory Medicine, Biomedical Research in Endstage and Obstructive Lung Disease Hannover (BREATH), German Center for Lung Research (DZL), Hannover Medical School, 30625 Hannover, Germany; janciauskiene.sabina@mh-hannover.de; 4 Department of Organic Chemistry, Faculty of Pharmacy, Ludwik Rydygier Collegium Medicum, Nicolaus Copernicus University in Toruń, Jurasza Str. 2, 85-089 Bydgoszcz, Poland

**Keywords:** cisplatin, meta-analysis, IC_50_, HeLa, HepG2, MCF-7, anticancer, cytotoxicity, antitumor, heterogeneity

## Abstract

Cisplatin is a classic anticancer drug widely used as a reference drug to test new metal complex drug candidates. We found an unexpected diversity in cisplatin-related cytotoxicity values, expressed as IC_50_ (the half-maximal inhibitory concentration) in tumour cell lines, such as MCF-7, HepG2 and HeLa. We reviewed the data published from 2018 to 2022. A total of 41 articles based on 56 in vitro experiments met our eligibility criteria. Using a meta-analysis based on a random effect model, we evaluated the cytotoxicity of cisplatin (IC_50_) after 48- or 72-h cell exposure. We found large differences between studies using a particular cell line. According to the random effect model, the 95% confidence intervals for IC_50_ were extremely wide. The heterogeneity of cisplatin IC_50_, as measured by the I^2^ index for all cancer cell lines, was over 99.7% at culture times of 48 or 72 h. Therefore, the variability between studies is due to experimental heterogeneity rather than chance. Despite the higher IC_50_ values after 48 h than after 72 h, the heterogeneity between the two culture periods did not differ significantly. This indicates that the duration of cultivation is not the main cause of heterogeneity. Therefore, the available data is diverse and not useful as a reference. We discuss possible reasons for the IC_50_ heterogeneity and advise researchers to conduct preliminary testing before starting experiments and not to solely rely on the published data. We hope that this systematic meta-analysis will provide valuable information for researchers searching for new cancer drugs using cisplatin as a reference drug.

## 1. Introduction

Cisplatin is widely used in everyday clinics as well as in clinical trials [1,2], and as a reference chemotherapeutic for the validation of new antineoplastic drugs and/or methods of treatment [3]. The main limitation of cisplatin is its high toxicity [4] and the risk of the development of intrinsic or acquired cancer cell resistance [5]. Some reports have linked cisplatin therapy with an increased risk of second cancers [6]. To overcome these problems, cisplatin is typically combined with other drugs or therapeutic methods [7,8,9,10]. Due to its simple structure and well-known pharmacological and toxicological profiles, cisplatin is also useful as a model drug in the search for new anticancer cures, especially for metal-based complexes, e.g., platinum-based complexes [11]. Specifically, cisplatin is applied as a reference drug for preliminary in vitro tests of potential new antitumor drugs.

Cytotoxicity studies in vitro are the first biological tests performed for potential new therapeutic substances. Cytotoxicity is a general term for how toxic a substance is to cells, and IC_50_ is a quantitative measure that specifies how much of a particular substance (e.g., a drug) is required to inhibit in vitro biological processes by 50%. In the context of cancer research, IC_50_ determines the concentration of a chemical compound that can inhibit cancer cell growth by half, relative to cells grown without the compound. IC_50_ is a very important measure that is also related to EC_50_, the plasma concentration required to obtain 50% of the maximum effect in vivo. Hence, the relationship between in vitro and in vivo cytotoxicity can help to reject chemical compounds during the initial stage of clinical study [12]. However, during data searching for our previous publication [11], we observed a diversity in the literature on the cytotoxic effects of cisplatin in cancer cell lines, i.e., in published IC_50_ values.

Primary cells and/or continuously growing cancer cells (cell lines) are primary in vitro models used for cytotoxicity tests. The cytotoxicity endpoint parameters include cell viability (e.g., trypan blue staining), cell membrane damage (e.g., LDH—lactate dehydrogenase assay), cell proliferation (e.g., Alamar Blue test), DNA damage (e.g., PCR (polymerase chain reaction), comet, halo, TUNEL (terminal deoxyribonucleotidyltransferase-mediated deoxyuridine triphosphate nick end labeling) assay, HPLC-electrospray tandem mass spectrometry, FISH (fluorescence in situ hybridization), FCM (flow cytometry), total protein content (sulforhodamine—SRB assay), mitochondrial function (including measurements of mitochondrial calcium, superoxide, mitochondrial permeability transition and membrane potential) or metabolic effects as indicators of the potential to cause toxicity to a cell culture e.g., MTT [3-(4,5-dimethylthiazol-2-yl)-2,5-diphenyl tetrazolium bromide] assays) [13,14]. We used some of the methods described below for the meta-analyses in this review.

The MTT test is a colorimetric assay that is most frequently used to determine cytotoxicity. It is based on the activity of succinate dehydrogenase, a mitochondrial enzyme of living cells that converts the soluble tetrazolium salt, 3-(4,5-dimethylthiazol-2-yl)-2,5-diphenyltetrazolium bromide, into its reduced form, insoluble formazan. The formazan crystal precipitates occur in small amounts or not in all damaged cells. To obtain reliable and reproducible results by using the MTT test, laboratory precision work is required at all stages of the test, particularly in the last stage of the assay, i.e., the dissolution of formazan crystals. Therefore, there many modifications to the MTT test have been introduced. One of these is CCK-8 assays, which use a tetrazolium salt as a substrate and which, under the influence of dehydrogenase, becomes converted into a colored, soluble compound instead of formazan crystals. In CCK-8 assays, a highly water-soluble tetrazolium salt is used; therefore, this test exhibits better detection sensitivity [15].

SRB assays allow for the determination of the total amount of protein in the examined sample, which is directly proportional to the number of cells. The basis of this method is the electrostatic binding of sulforhodamine to proteins at an appropriate pH, depending on the qualitative composition of amino acids, after cell fixation with trichloroacetic acid [16].

The assay of intracellular ATPs allows for the determination of the efficiency of mitochondrial energy processes, which reflect cell viability. The change in the ATP is proportional to the increase or decrease in the number of cells, as well as to the decrease in the efficiency of energy processes in cells. The determination of the number of ATPs can be based on bioluminescence occurring in the reaction with the luciferase catalyzing the oxidation of luciferin to oxyluciferin, with the participation of one ATP molecule [17].

In this review, we have focused on the publications describing new anticancer compounds (mainly metal complexes), in which cisplatin was used as a reference drug. Cell lines like HeLa, HepG2, and MCF-7, selected as the subject of this study, are the most frequently used in cytotoxicity studies. Some published results have shown differences in the IC_50_ values of carboplatin, etoposide, paraquat in an in vitro model based on human glioblastoma cells [18]. Our aim was to investigate the reliability and reproducibility of cisplatin cytotoxicity in selected cancer cell lines (HeLa, HepG2 and MCF-7) based on the analysis of published data for 2018–2022 years in available databases such as Science Direct, Scopus, and PubMed. The possible reasons of the heterogeneity in results was also of interest.

## 2. Results

Source data for three cell lines are given in Files S1–S3 (Tables SA1–SC1), which contain averaged IC_50_ values, and for HepG2 or MCF-7 lines, IC_50_ values are given separately for 48 and 72 h cell culture. The results of IC_50_ effects examined via meta-analysis are presented in detail in the subsections below. The deviance from averages is given in Files S1–S3. In all tables, for the numbers between −1 and 1, the zeros preceding decimal dots are omitted.

### 2.1. Cytotoxicity of Cisplatin in HeLa Cell Cultures

HeLa was the first human cell line derived from Henrietta Lacks aggressive adenocarcinoma of the cervix in 1951 [19]. Currently it is one of the most used human cell lines for the search of new anticancer compounds [20,21]. According to our observations during the review of publications, a 48 h culture of HeLa cells is most often used for in vitro testing [11]. Table SA1 (File S1), shows the IC_50_ raw values of cisplatin after 48 h of detailed described cell cultures in fourteen publications.

**Figure 1:**
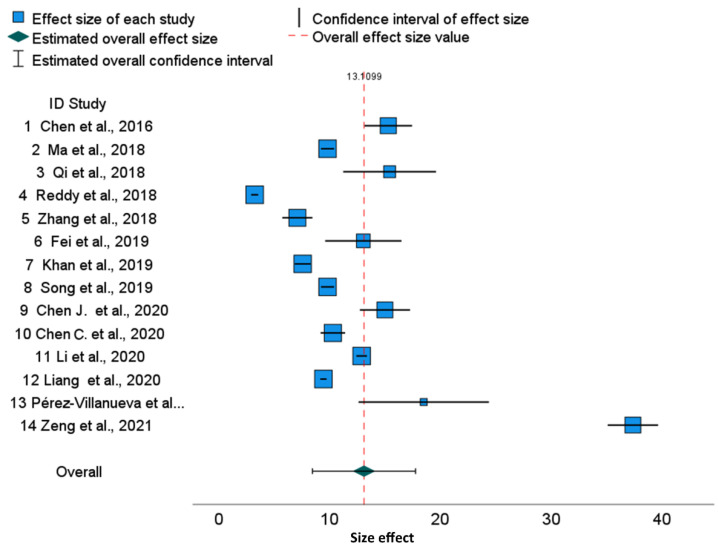
Cisplatin IC_50_ in 48 h HeLa cell cultures. Effects for individual studies with overall effect. Forest plot [23,24,25,26,27,28,29,30,31,32,33,34,35,36].

The cytotoxicity data of cisplatin in HeLa cells cultured for 48 h after meta-analysis for the mixed effects model is presented in Figure 1 in the form of a forest plot.

In this graphical representation of the meta-analysis results, each row represents the results of an individual study. Blue boxes represent the individual studies, with their size reflecting the weights (estimated by the inverse-variance) or relative weights (Table 1). The corresponding whiskers represent 0.95 confidence intervals for IC_50_ which quantify the uncertainty in the corresponding point estimates (Table 1 and Table 2). The green diamond and red-dotted vertical line represent the overall effect (13.1099). Estimated overall 0.95 confidence intervals are visible as horizontal segments adjacent to the diamond. The detailed values are given in Table 1 and Table 2. A strong inconsistency of IC_50_ values can be noticed (Figure 1), and many 0.95 confidence intervals are disjoint (please compare with values displayed in Table 1).

When assessing the reliability of the data, it is important to consider the heterogeneity of the studies. To assess this heterogeneity, the following statistics were used: Q, τ^2^, H^2^, and I^2^ [22] (Table 3, Table 4 and Table 5). The higher statistical values of mentioned statistics are directly related to the greater heterogeneity of the study (see in Section 4.3 for details). Analysis based on Cochran Q statistics suggests rejecting the hypothesis of homogeneity (*p* < 0.0005) (Table 3). The I^2^ index is a transformation of H (where H^2^ = 536.13) that describes the part of the total variation. It is assessed as I^2^ = 1 − 1/H^2^ = 99.99%, which is extremely high, showing that variability among effect sizes is caused not by sampling error but by true heterogeneity between studies. High I^2^ means that IC_50_ values are inconsistent between publications. From the point of view of the forest plot (Figure 1), I^2^ reflects overlaps between individual confidence intervals overlap.

**Table 1:** Cisplatin IC_50_ in 48 h HeLa cell cultures. Effect size estimates for individual studies.

ID	Study	Effect Size	Std. Error ^a^	t	95% Confidence Interval		Weight	Weight (%)	Ref.
					Lower	Upper			
1	Chen et al., 2016	15.300	1.0970	13.948	13.150	17.450	0.015	7.2	[23]
2	Ma et al., 2018	9.820	0.3002	32.709	9.232	10.408	0.016	7.3	[24]
3	Qi et al., 2018	15.420	2.1362	7.218	11.233	19.607	0.015	6.8	[25]
4	Reddy et al., 2018	3.250	0.1617	20.104	2.933	3.567	0.016	7.3	[26]
5	Zhang et al., 2018	7.100	0.6928	10.248	5.742	8.458	0.016	7.3	[27]
6	Fei et al., 2019	13.050	1.7551	7.435	9.610	16.490	0.015	7.0	[28]
7	Khan et al., 2019	7.580	0.3667	20.670	6.861	8.299	0.016	7.3	[29]
8	Song et al., 2019	9.820	0.3002	32.709	9.232	10.408	0.016	7.3	[30]
9	Chen J. et al., 2020	15.000	1.1547	12.990	12.737	17.263	0.015	7.2	[31]
10	Chen C. et al., 2020	10.300	0.5650	18.230	9.193	11.407	0.016	7.3	[32]
11	Li et al., 2020	12.900	0.2449	52.664	12.420	13.380	0.016	7.3	[33]
12	Liang et al., 2020	9.450	0.1443	65.472	9.167	9.733	0.016	7.3	[34]
13	Pérez-Villanueva et al., 2021	18.500	3.0000	6.167	12.620	24.380	0.014	6.4	[35]
14	Zeng et al., 2021	37.370	1.1605	32.202	35.096	39.644	0.015	7.2	[36]

^a^ Truncated Knapp–Hartung method is used for SE adjustment.

**Table 2:** Cisplatin IC_50_ in 48 h HeLa cell cultures. Overall effect size estimate.

	Effect Size	Std. Error ^a^	t	95% Confidence Interval		95% Prediction Interval ^b^	
				Lower	Upper	Lower	Upper
Overall	13.110	2.1523	6.091	8.460	17.760	−4.860	31.079

^a^ Truncated Knapp-Hartung method is used for SE adjustment. ^b^ Based on t-distribution.

**Table 3:** Cisplatin IC_50_ in 48 h HeLa cell cultures. Test of homogeneity.

	Chi-Square (Q Statistic)	df	Sig.
Overall	2194.156	13	<0.0005

Df—degrees of freedom, Sig.—significance.

**Table 4:** Cisplatin IC_50_ in 48 HeLa cell cultures. Heterogeneity measures

Time	Measure	Value
Overall (48 h)	Tau-squared	63.386
	H-squared	536.126
	I-squared (%)	99.8

**Table 5:** Cisplatin IC_50_ in 48 HeLa cell cultures. Egger’s regression-based test ^a^.

Parameter	Coefficient	Std. Error	t	Sig. (2-Tailed)	95% Confidence Interval	
					Lower	Upper
(Intercept)	9.117	2.9985	3.040	0.010	2.584	15.650
SE ^b^	4.410	2.4856	1.774	0.101	−1.006	9.825

^a^ Random effects meta-regression with the Truncated Knapp–Hartung SE adjustment. ^b^ Standard error of effect size.

Prediction intervals are much wider than confidence intervals (Table 2) due to the large heterogeneity τ^2^. In summary, we conclude that the results of included studies are heterogenic and not consistent. Additionally, publication 14 is an outlier study.

The regression of Egger’s test for funnel plot asymmetry is significant (Table 5; *p* = 0.01). Hence, in agreement with the forest plot, it shows the asymmetry of the results.

Additionally, the differences between each published IC_50_ value from the averaged IC_50_ values were examined and the results are presented in File S1 (Table SA2 and Table SA3 and Figure SA1). The confidence interval not reaching the vertical zero line suggests significant deviance from averaged IC_50_, which is confirmed in File S1, Table SA2. Out of 14 examined publications, 9 numbered 2, 4, 5, 7, 8, 10, 12, 13, and 14 are significantly different from IC_50_ average for cisplatin IC_50_ after 48 h in HeLa cell cultures (File S1, Table SA2, Figure SA1).

### 2.2. Cytotoxicity of Cisplatin in HepG2 Cell Cultures

The human hepatoma HepG2 cell line is widely used as an in vitro model of the human liver. These cells display a high degree of morphological and functional differentiation of liver cells in vitro and are easy to handle. Therefore, HepG2 is another frequently used tumor cell line derived from human hepatocellular carcinoma (HCC) [37]. The mechanism of HepG2 cell acquisition and resistance may be related to cell-derived exosomes [38]. The exosomes are the small vesicles (30–150 nm) released by many cell types. The vesicles may contain lipids, proteins, and nucleic acids from host cells, and can be transported by body fluids (e.g., lymph, saliva, blood, cerebrospinal fluids, urine) through the tissues. Interestingly the exosome’s membrane constitutes a stabile barrier and helps to protect the contains from enzymatic degradation. Due to this property, the exosomes are involved in many important processes, e.g., cell-to-cell communication, transport of some factors, or immunoregulation. During the HCC, exosomes can create a suitable microenvironment for tumor growth via effects on signal pathways and angiogenesis. Additionally, exosomes can enhance metastasis by epithelial–mesenchymal transformation, extracellular matrix degradation, and vascular leakage [39].

**Figure 2:**
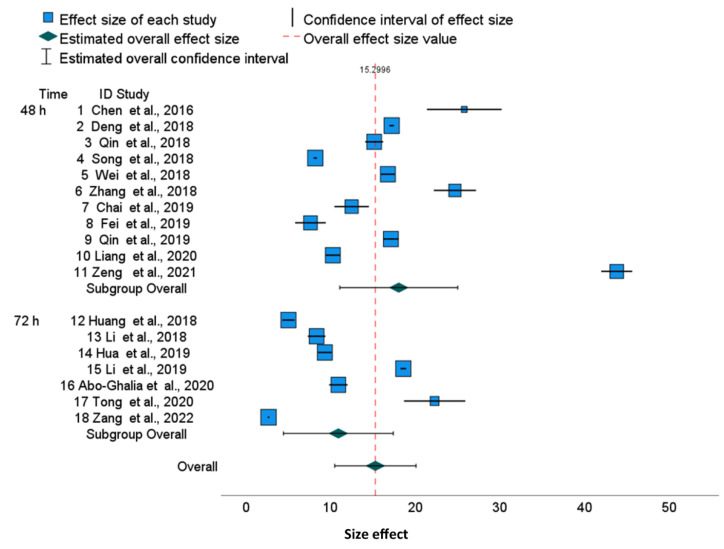
Cisplatin IC_50_ in 48 and 72 h HepG2 cell cultures with subgroups and overall effect size-forest plot [23,27,28,34,36,40,41,42,43,44,45,46,47,48,49,50,51,52].

The raw data and details of cell culture in 18 publications are presented in Table SB1, (File S2) among them, 11 are based on 48 h cell culture and 7 on 72 h cell culture. Figure 2 illustrates the cytotoxicity values of cisplatin after 48 h and 72 h of HepG2 culture and the red dashed vertical line shows the overall effect for all publications. Data are presented as 0.95 confidence intervals in the forest plots for each individual study effect and as the summary of two groups, both joined and adjusted for different experimental conditions, i.e., 48 h and 72 h (Figure 2).

Detailed values of lower and upper bounds of confidence intervals for each publication and overall effect (with subgroup effects) are presented in Table 6 and Table 7, respectively. Within the time-subgroups, most of the 0.95 confidence intervals are disjoint (horizontal segments in Figure 2) for the effect of cisplatin. Green diamonds represent an effect of a time-subgroup with a confidence interval of 0.95 denoted as a horizontal segment. The last green diamond and the red-dotted vertical line represent the overall effect (15.2996). Overall, 0.95 confidence intervals are presented as horizontal segment adjacent diamonds. Summary results of meta-analyses in the forest plot (Figure 2) illustrate significant heterogeneity.

The confidence interval around the variable effect depends on the variance between the studies τ^2^ = 106.532 (for 48 h) and smaller τ^2^ = 47.775 (for 72 h) and the individual standard errors. We noticed a large discrepancy between IC_50_ in publications, although they are slightly smaller for 72 h than for 48 h [CI 95% 4.44; 17.41 for 72 h vs. 11.10; 25.03 for 48 h].

Table 8, Table 9 and Table 10 show statistics Q, τ^2^, H^2^, and I^2^ for 48 h and 72 h, which assess the heterogeneity of the studies. Homogeneity analysis based on Q statistics rejects the hypothesis of homogeneity in both culture duration subgroups and for a joint set of papers (*p* < 0.0005) (Table 8). The variance in the observed effects’ τ^2^ values depends on the effect size of cisplatin cytotoxicity on HepG2 cells that is higher after 48 h (106.532) than after 72 h (47.775). However, the variance value depends on the effect size (cytotoxicity measured by IC_50_ for HepG2), which is also higher after 48 h. Thus, the square roots of variances relative to means, i.e., variability coefficients are similar: 106.532^0.5^/18.07 = 0.57 at 48 h and 47.775^0.5^/10.93 = 0.63 at 72 h. Moreover, the results of the Q Cochran test of inter-group homogeneity were not significant (*p* = 0.08; Table 9).

The statistical analysis presented in Table 10 confirms the considerable heterogeneity. The H^2^ statistic is larger for a subgroup of 48 h (1095.3) than for a 72 h (784.2), and the overall H^2^ is 1947.6. Inconsistency in the findings among the included studies based on experimental times was also compared by the I^2^ index. For example, according to Table 10, this statistic for 48 h (11 studies), I^2^ = (H^2^ − 1)/H^2^ = 1 − 1/1095.319 = 99.91%, indicates significant heterogeneity. Similarly, we observed significant heterogeneity for 72 h culturing (7 studies), according to index I^2^ = 1 − 1/784.171 = 99.87%, and significant overall heterogeneity according to I^2^ = 1 − 1/1947.583 = 99.94%. The prediction intervals are much wider than confidence intervals (Table 7) due to the significant heterogeneity τ^2^, both for all publications and for in the publication subgroups. Thus, significant inconsistency exist among the raw data even after separation into different experimental times of HepG2 cells culture.

According to the regression Egger test for funnel plot asymmetry (Table 11), there is no significance for 72 h (*p* = 0.089) but for 48 h (*p* = 0.032). The higher asymmetry after 48 h is due to the outlier study (numbered 11).

To finish, we calculated differences between cisplatin IC_50_ and averaged values for IC_50_ assessed from 11 publications for 48 h and from 7 publications for 72 h based on the HepG2 cell line. Almost all individual effects with 95% confidence intervals did not reach zero (Figure SB1, File S2), which is concordant with the fact that the deviation is significant (*p* < 0.001) for all examined publications except for the 72 h study numbered 16 (*p* = 0.853; Table SB2, File S2). Additionally, we can see an outlier study for 48 h (numbered 11).

### 2.3. Cytotoxicity of Cisplatin in MCF-7 Cell Cultures

**Figure 3:**
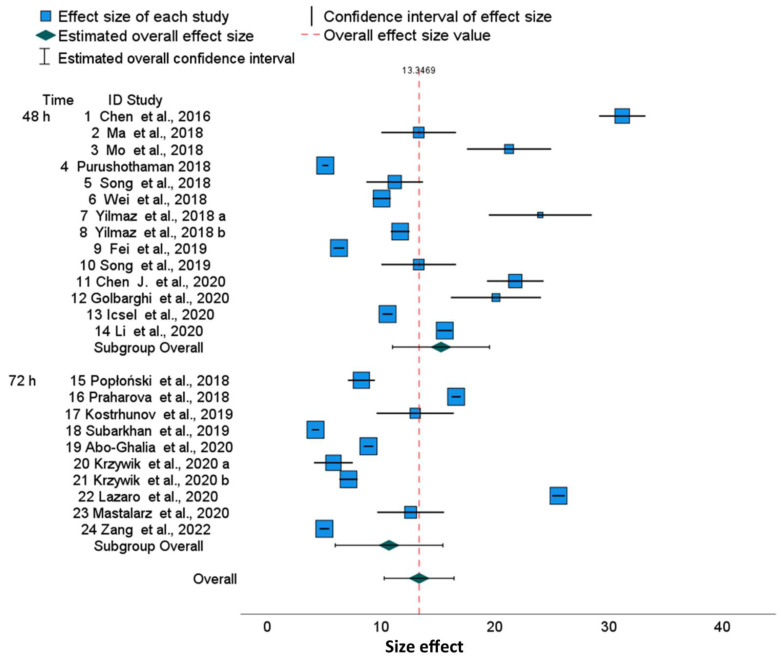
Cisplatin IC_50_ in 48 and 72 h MCF-7 cell cultures with subgroups and overall effect size- forest plot [23,24,28,30,31,33,42,43,52,54,55,56,57,58,59,60,61,62,63,64,65,66,67].

MCF-7 is another commonly used human breast cancer cell line for new anticancer drug search in breast cancer (Table 12) [53]. The cytotoxicity of cisplatin in 48 and 72 h of MCF-7 culturing is shown in the forest plot (Figure 3).

Horizontal segments adjacent to blue boxes show (Figure 3) that most of the 0.95 confidence intervals are disjoint for cisplatin effects. The green diamonds for 48 and 72 h represent time-subgroup effects with 0.95 confidence intervals represented as corresponding horizontal segments. The last green diamond on the forest plot and the red-dotted vertical line represents overall effect (13.3469). Corresponding estimated overall 0.95 confidence intervals (for individual publications concerned with MCF-7) are represented as horizontal segments adjacent to the diamond.

In these analyses, we have again observed considerable inconsistency in the raw data under different time points of MCF-7 cell culture (Table 12 and Table 13, Figure 3). The hypothesis for homogeneity is rejected according to Cochran Q statistics (Table 14) both for the 48 and 72 h subgroups and for the joint set of publications (*p* < 0.0005). τ^2^ equal to 52.84 is the variance of the observed effects of 48 h culturing, while the variance τ^2^ for 72 h is lesser: 43.14 (the overall value is 51.61).

The square roots of variances relative to means, i.e., variability coefficients are 52.84^0.5^/15.27 = 0.47 at 48 h and 43.14^0.5^/10.70= 0.61 at 72 h.

Again, based on the Cochran Q test comparing subgroup variances, the subgroup homogeneity test is not significant (Table 15, *p* = 0.112). As shown in Table 16, I^2^ = 99.7% at 48 h indicates significant heterogeneity. Likewise, there is significant heterogeneity at 72 h (I^2^ = 99.8%) as well as an overall heterogeneity of I^2^ = 99.8%. The prediction intervals are much wider than the confidence intervals due to the substantial τ^2^ variance (Table 13).

Due to the results showing heterogeneities using MCF-7 cells, the findings in existing studies are inconsistent. Additionally, for the study numbered 1, one can observe the most distant value (Figure 3). The Egger’s regression test for funnel plot asymmetry is significant for 48 h (*p* = 0.007), for 72 h (*p* = 0.015) and for a joint group (*p* < 0.001) (Table 17). Figure SC1, App. C displays the results of meta-analysis for differences with subgroup averages. The grey, solid vertical line (x = 0) divides the graph into two parts: a right side representing articles with an IC_50_ effect higher than the subgroup average and a left side- for articles with IC_50_ effect smaller than subgroup average. The grey, solid vertical line (x = 0) is distant (by −1.84791) from a red-dashed line which denotes overall differences. The differences are not observed for HeLa and HepG2 lines, where the grey and red lines almost overlap. In fact, for the MCF-7 cell line, a larger variety of experimental methods was used as compared to other lines (Table SC2, File S3). The differences between IC_50_ and the averaged values for IC_50_ are significant for 11 out of 14 publications after 48 h and for 8 out of 10 publications after 72 h of cell culture (Table SC2, Figure SC1, File S3).

## 3. Discussion

The phenomenon of the same drug differences in IC_50_ cytotoxicity in the same cell line in vitro has already been described in previous studies [18,68]. Among the reasons for this diversity are cell density, cell culture time, and the method of cytotoxicity detection [68]. To investigate further the variability of published results, we analyzed IC_50_ values of cisplatin in three human cancer cell lines (HeLa, HepG2 and MCF-7). The obtained results were divided according to the duration of the cell culture time. Among the analytical methods determining the IC_50_ value, the MTT test was a dominant, while the SRB, ATP, and CCK-8 methods were also used (Tables SA1, SB1 and SC1 in Files S1–S3); the methods were briefly described in the Introduction.

The forest plots illustrate a large discrepancy between cisplatin IC_50_ values. From many factors putatively impacting results, only cell culture time and analytical methods determining the IC_50_ values are available in publications. Therefore, to perform a more homogenous evaluation of the collected publications, we sub-grouped them according to cell culture time. The 95% confidence intervals for specific cell lines used in different publications are disjoint, which results in significant differences between published results. This also revealed that, for various tumor lines, the range of IC_50_ joined via meta-analysis is slightly different, suggesting that some cell lines are more resistant to cisplatin than others. For example, in the HepG2 line, cisplatin cytotoxicity after 48 h is 18.07 (95% CI, 11.10–25.03) and after 72 h, it is 10.93 (95% CI, 4.45–17.41), which shows that the confidence intervals in subgroups are overlapping. Similarly, we can show the overlapping of 95% confidence intervals for MCF-7 cells after 48 h [15.27 (95% CI 11.01–19.53), and after 72 h, it is 10.70 (95% CI, 6.00–15.44)], whereas the overall cytotoxicity is 13.38 (95% CI, 10.28–16.42) (Figure 3). Finally, the overall cytotoxicity of the cisplatin in HeLa cell line after 48 h is 13.11 (95% CI, 8.46–17.76) (Figure 1). Thus, the differences described above are not substantial between cell lines as well as between cell culture times, because the confidence intervals are not disjoint. The reason is the substantial diversity within three cell lines and within culture time subgroups. The I^2^ indices which describe the proportion of total variation across studies are over 99.8% for HeLa and HepG2 and over 99.7% for MCF-7. Independently on the cell culture time, the values of I^2^ are extremely high. Thus, for all examined cell lines, the heterogeneity cannot be attributed to cisplatin exposure time only. Furthermore, for all cell lines, overall and within time-subgroups, the results of the Q Cochran test for homogeneity were significant (*p* < 0.0005). Notwithstanding, the significance of the Cochran Q test comparing variances after 48 h and 72 h was not obtained, confirming that this heterogeneity is not related to the cell culture time. Similarly to I^2^ indices, other statistics measuring the heterogeneity such as τ^2^ and H^2^ confirm substantial heterogeneity within time-subgroups and within the overall effect. The prediction intervals are much wider than confidence intervals due to the substnatial heterogeneity τ^2^, both for all publications and for the overall effects in the time-subgroups.

As confirmed by the asymmetry forest plots, most of the results of the Egger’s regression tests for funnel plot asymmetry are significant (e.g., HeLa, MCF-7 overall, and within 48 and 72 h subgroups and for HepG2 during the 48 h culture). Moreover, 0.95 confidence intervals for differences between individual IC_50_ values and the averaged values within time-subgroup IC_50_ illustrate significant deviations from these averaged values in most cases.

To the best of our knowledge, there are few publications that attempt to describe the quality of the IC_50_ results. In the work of Damian et al. [18], the IC_50_ values of three different anticancer drugs: carboplatin, etoposide, and paraquat were tested against two glioblastoma lines—U87MG and U373MG—via the four analytical methods simultaneously (acid phosphatase, MTT, Almar Blue, and trypan blue). Different IC_50_ values were obtained for each reference drug depending on the method used. For the cytotoxicity values determined using trypan blue, significant differences were observed compared to other methods. The obtained results allowed to the researchers to distinguish the advantages and limitations of each of the tested methods [18].

In a more recent work by Arokia Femina et al. [68], the IC_50_ values of 5-fluorouracil tests available in the literature on 10 types of human cancer cell lines (AGS (gastric adenocarcinoma), DLD1 and SNU-C4 (colorectal adenocarcinoma), HCT116 (colorectal carcinoma), HT-29 and MKN28 (gastric adenocarcinoma), MKN45 and SGC7901 (gastric carcinoma), SK-MES-1 (lung carcinoma), SW620 (colon adenocarcinoma)) were compared by using MTT, CCK-8, SRB, and a clonogenic assay in 12–72 h cultures with different cell densities. A wide scatter of the 5-fluorouracil IC_50_ results (values 1.46–289.7 µM) was observed. According to the authors of the study, these differences may be related to the type and proliferative potential of the cell line used, the method used, the seeding density, the drug exposure time, and its concentration.

Our research has focused on one of the most used anticancer drugs—cisplatin—which is the reference drug for many newly developed complexes with antiproliferative activities. Contrary to earlier authors, we conducted a broad review of the literature and ranked the results according to the three cell lines and the cultivation time. For the two tested HeLa and HepG2 lines, almost all results were obtained via the MTT method; only in the case of MCF-7 was the variety of methods used greater. The wide spread of IC_50_ values obtained for the HeLa (48 h) and HepG2 (48 h and 72 h) lines indicates a significant influence of factors other than the type of cell line, the duration of culture, and the method used. In our research, we did not consider the effect of cell culture density, although the condition of the tested cell line and the preparation of the drug may have an impact. When new complex compounds are tested, there is often a problem with their solubility, so a certain amount of solvent, e.g., dimethyl sulfoxide (DMSO) is added to the culture. The methodology of the work rarely mentions whether the same amount of the same solvent was added to the control or the reference drug culture. According to our results, the large overall heterogeneity is not due to different cell culture times, but is due to other factors that are difficult to determine from published studies.

Authors typically do not provide enough information about experimental conditions that might help to explain inconsistency within the findings. Furthermore, the quality of experimental data description differs among publications. The observed diversity of results might also be related to the quality of tested cancer cell lines. In different research centers, cancer cell lines, despite having the same origin, may have their own “story”; in particular, such as the cell culture’s passage, the pre-assay preparation of the cell line and reagents, the concentration of cells, and the cell culture medium, among others. Interestingly, in 2003, the report from Deutsche Sammlung von Mikroorganismen und Zellkulturen (DSMZ) reported significant contamination of cell lines. About 18% of 252 “new” hematopoietic cell lines were cross-contaminated (by other cell lines). The scale of this problem emphasized the value of good laboratory practice [69]. Additionally, it would be helpful to provide some functional tests for used cell lines like free radicals and cytokine/chemokine production after exposure to cytotoxic drugs. Moreover, the methods and protocols need to be considered, e.g., colored compounds could interfere in a test with absorbance measurements.

On the other hand, determining the IC_50_ values of cisplatin might be not the aim of a study; this value is often presented only for comparison with a chemical compound of interest in tumor treatment. Nevertheless, unexpected inconsistency in results makes it questionable that we have reliable models to test new anti-cancer drugs’ cytotoxicity in vitro.

Further publications providing a better description of experimental conditions may help to determine the main factors affecting results and help to clarify the reasons for the inconsistency of published results.

## 4. Materials and Methods

### 4.1. Data Selection

Meta-analysis was performed according to the PRISMA (Preferred Reporting Items for Systematic Reviews and Meta-Analyses) statement guidelines [70].

#### 4.1.1. Databases SEARCH Criteria

A combination of the key words, IC_50_, cytotoxicity, anticancer, antitumor, cisplatin, HeLa, HepG2, or MCF-7 cells was used. Databases searched: Science Direct, Scopus, and PubMed, for full-text research articles published between 2018 and 2022, and only journals in the field of chemistry, pharmacology, toxicology, or pharmaceutical science.

#### 4.1.2. Records Identified

When searching Science Direct, n = 2817; Scopus, n = 2431; and PubMed, n = 691.4.1.3. Eligibility:

Records excluded as duplicates (n = 2925) and for other reasons (n = 2974). The reasons for excluding full text articles were as follows:

1. Cisplatin IC_50_ results in cell lines other than HeLa, HepG2, or MCF-7;
2. Cisplatin IC_50_ data for cell culture times other than 48 and 72 h;
3. Results obtained under unusual or special conditions (e.g., light irradiation in photodynamic therapy) or under culture conditions described as ambiguous;
4. Cisplatin IC_50_ results obtained using 3D spheroid cultures.

### 4.2. Studies Included in the Meta-Analysis

Forty studies were included in meta-analyses, based on the eligibility criteria. Of these studies, four reported results were based on MCF-7 and HepG2 cells, one was based on MCF-7 and HeLa cells, and three were based on HeLa and HepG2 cells. Only one publication reported results based on all three cell lines. To enhance results, a study published in 2016 describing cisplatin IC_50_ values for all three cancer cell lines was included.

### 4.3. Statistical Methods

Of interest was the reliability of the IC_50_ results for a specific cell line and for a specific culture period. In the same cell culture, cisplatin has a different IC50 value at 48 and 72 h; therefore, the effect of this drug may differ in studies with different experimental times. Cell culture time is a potential factor for cytotoxicity and was taken into account when performing the meta-analysis. For either HepG2 or MCF-7 cancer lines, each article published the results for one culture time point of interest i.e., 48 h or 72 h. In these cases, a meta-analysis was performed for two independent subgroups within the studies (48 h and 72 h). However, time subsets were not available for HeLa cell lines, as the vast majority of results were found for a culture time of 48 h. In order to examine whether the different IC_50_ values were due to real differences (heterogeneity), or whether the diversity of the IC_50_ results occurred by chance (homogeneity), the heterogeneity statistics of the cisplatin effects measured via IC_50_ were examined via meta-analysis. Measures of effect heterogeneity indicate the extent to which the differences between results of individual studies influenced the overall effect. The heterogeneity analysis was carried out separately for each time group.

Cochran’s homogeneity Q statistics are the weighted sum of squared differences between individual study effects and the pooled effect across studies, with weights the same as for the pooling method. Homogeneity was analysed by testing whether the variability between studies τ^2^ was equal to zero. This was based on Cochran’s homogeneity test Q statistic with *p*-value based on a chi-square distribution with *k*-1 degrees of freedom (*k* is the number of studies). The Q test for homogeneity hypothesis was used to obtain information about the presence or absence of heterogeneity (e.g., absence of heterogeneity if the test is non-significant). However, to report on the extent of this heterogeneity, other statistics were used. For example, the heterogeneity was assessed using the inter-study variance τ^2^. Additionally, I^2^ and H^2^ indices were calculated to assess heterogeneity [71]. I^2^ = (H^2^ − 1)/H^2^ expresses the amount of variability in a meta-analysis that is explained by the inter-trial heterogeneity rather than sampling error. Unlike *Q*, it does not necessarily depend on the number of studies included in the meta-analysis. I^2^ index can be directly compared between meta-analyses with different numbers of studies and different types of outcome data [72].

Because the studies chosen in the meta-analysis were from different sites and likely included results of different specifications (e.g., different methods of obtaining IC_50_), the random effects model was chosen, which assumes that there are meaningful differences between studies.

The estimation of the effect is achieved by the iterative method of computing the restricted maximum likelihood estimate (REML).

The truncated Knapp–Hartung method [73] (truncates the value if it is less than 1 when estimating the variance–covariance matrix) was used to adjust the standard error. According to the published recommendations, Hartung–Knapp method for random effect meta-analysis provides more accurate error rates than the DerSimonian and Laird method, especially for only a few studies [74]. This method is also recommended when the accuracies of the studies vary [75].

Lower and upper bounds of the confidence intervals for individual publications and overall effect including subgroup effects were evaluated. Forest charts were added to illustrate the summary of results of meta-analyses and to give a visual impression of the degree of the heterogeneity of the studies.

Random effect weights were estimated using the inverse variance, including within-study *SE_i_*^2^ and inter-study variance. The weight *w_i_* of the study depends on the observed variability according to the formula:

$$w_{i}=\frac{1}{SE_{i}^{2}+\tau^{2}}$$

where *SE_i_* is the standard error within each study and *τ*^2^ is the variance between the studies.

The variability in the obtained effects for each study is due to the sampling error *SE_i_* and the differences between the study populations *τ*^2^. Weights *w_i_* or relative weights *w_i_*/∑*w_i_* define the size of the squares in the forest plot. The random effect estimates a weighted average of the impact of each publication. The confidence interval for the effect depends on *τ*^2^.

In meta-analysis, it is important to assess the bias. Publication bias appears because studies with desirable results are more likely to be published. Consequently, published results may be biased in a certain direction. Analysis of the publication bias was performed using the Eggers’ regression-based test for meta-analysis with continuous outcomes [76,77]. The Egger’s test for asymmetry was performed by examining the linear regression of the standardized effect (*e*/*SE*) on the precision (1/*SE*):

$$\frac{e}{SE}=\alpha +\frac{\beta }{SE}+\varepsilon$$

where *e* is the estimated true effect, *SE* is the standard error of effect, and *ε* is a random noise. The size of *α* (intercept) indicates the extent of the asymmetry. Eggers’ test estimates the statistics based on the t-distribution. Test of intercept *α* = 0 is based on *t*-distribution with *k*-2 degrees of freedom. Additionally, to test for publication bias in the meta-analysis, a trim-and-fill analysis was applied (results not presented).

In addition, to assess the significance of the deviation from the mean value for a specific publication (also the 48 h and 72 h time subgroups), for each tumor cell line, and culture time considered, the difference between individual published IC_50_ values and averaged IC_50_ values for the respective time were calculated. It was assumed that for a specific cell line (HeLa, HepG2, MCF-7) and for a specific culture time (48 or 72 h), the correct IC_50_ value is approximated by the average of the relevant values.

Each individual difference can be viewed as an individual effect. Thus, the effect, ${\tilde{e}}_{i}$, is defined as the difference between the *i*-th individual published IC_50_ value and averaged IC_50_ value

$${\tilde{e}}_{i}=m_{i}-m\;\mathrm{for}\;i=1,\ldots ,k$$

where *k* is the number of publications, as the particular time of cell culture (48 or 72 h); *m_i_* is the individual published IC_50_, and *m* is the assumed theoretical IC_50_ assessed as the average of *m_i_*. In addition, corresponding standard errors ${\tilde{SE}}_{i}$ were calculated from source data, i.e., *SD_i_* and *n_i_* (given in Tables SA1, SB1 and SC1 in Files S1–S3). The meta-analysis for deviances is given in numerical tables and corresponding forest diagrams. The prediction intervals are also presented in order to reflect the expected uncertainty in the summary effect when a new study was added to the meta-analysis. Prediction intervals for substantial heterogeneity τ^2^ are much broader than confidence intervals.

The PS IMAGO PRO 9.0 package was used to create tables and figures in meta-analyses subsections. PS IMAGO PRO is an integrated tool for performing tasks in the field of statistical data analysis [78]. Graphs were created using default options in the meta-analysis for numerical variables.

## 5. Conclusions

In 42 studies published between 2018 and 2022, we found an unexpected degree of diversity in cisplatin-related cytotoxicity values in MCF-7, HepG2, and HeLa tumour cell lines. After performing a meta-analysis using mixed-effect models, we observed a substantial degree of heterogeneity in the cisplatin cytotoxicity effects assessed via I^2^ indices at the 99.8% level in the HeLa cell line, at 48 h culturing. For a single cancer cell line such as HepG2 and MCF-7, splitting the data by cell culture times (48 and 72 h) resulted in the same degrees of diversity, as measured via I^2^. This indicates that experimental duration is not the main cause of this inconsistency. A substantial degree of heterogeneity was confirmed by other statistics such as τ^2^, H^2^, and the significant Q Cochran test for homogeneity. For all cancer cell lines considered, the differences between individual publications and the deviation of the IC_50_ values from the means of the time-subgroup values were often significant.

To determine the reasons for such diversity in the published results, the stratified analyses of a large series of reports with comprehensive descriptions of their experiment conditions would be helpful. Many factors can affect the quality of cytotoxicity test results, including cell line quality, study protocol validation, and the optimal selection of techniques.

The observed inconsistency in reported cytotoxicity results reduces confidence when comparing new compounds with published cisplatin IC_50_ values. The data available in the literature are too diverse and unreliable to serve as a reference. It is therefore advisable to carry out a separate reference control for each new experiment, and to not rely solely on the available literature data of IC_50_.

## Figures and Tables

**Table 6:** Cisplatin IC_50_ in 48 and 72 h HepG2 cell cultures. Effect size estimates for individual studies.

	ID	Study	Effect Size	Std. Error ^a^	t	95% Confidence Interval		Weight	Weight (%)	Ref.
						Lower	Upper			
48 h	1	Chen et al., 2016	25.800	2.2517	11.458	21.387	30.213	0.010	5.3	[23]
	2	Deng et al., 2018	17.230	0.1386	124.347	16.958	17.502	0.011	5.6	[40]
	3	Qin et al., 2018	15.160	0.5411	28.016	14.099	16.221	0.011	5.6	[41]
	4	Song et al., 2018	8.200	0.0981	83.546	8.008	8.392	0.011	5.6	[42]
	5	Wei et al., 2018	16.780	0.4164	40.297	15.964	17.596	0.011	5.6	[43]
	6	Zhang et al., 2018	24.700	1.2702	19.446	22.211	27.189	0.011	5.5	[27]
	7	Chai et al., 2019	12.500	1.0392	12.028	10.463	14.537	0.011	5.5	[44]
	8	Fei et al., 2019	7.630	0.9238	8.260	5.819	9.441	0.011	5.6	[28]
	9	Qin et al., 2019	17.130	0.4696	36.480	16.210	18.050	0.011	5.6	[45]
	10	Liang et al., 2020	10.280	0.4446	23.124	9.409	11.151	0.011	5.6	[34]
	11	Zeng et al., 2021	43.810	0.9353	46.840	41.977	45.643	0.011	5.6	[36]
72 h	12	Huang et al., 2018	5.030	0.3695	13.613	4.306	5.754	0.011	5.6	[46]
	13	Li et al., 2018	8.360	0.5312	15.739	7.319	9.401	0.011	5.6	[47]
	14	Hua et al., 2019	9.330	0.4157	22.444	8.515	10.145	0.011	5.6	[48]
	15	Li et al., 2019	18.600	0.1732	107.387	18.261	18.939	0.011	5.6	[49]
	16	Abo-Ghalia, 2020	10.930	0.5554	19.679	9.841	12.019	0.011	5.6	[50]
	17	Tong et al., 2020	22.300	1.8475	12.070	18.679	25.921	0.010	5.4	[51]
	18	Zang et al., 2022	2.680	0.0490	54.705	2.584	2.776	0.011	5.6	[52]

^a^ Truncated Knapp–Hartung method was used for SE adjustment.

**Table 7:** Cisplatin IC_50_ in 48 and 72 h HepG2 cell. Effect size estimates for subgroup analysis.

	Effect Size	Std. Error ^a^	t	95% Confidence Interval		95% Prediction Interval ^b^	
				Lower	Upper	Lower	Upper
48 h	18.066	3.1266	5.778	11.100	25.032	−6.330	42.462
72 h	10.929	2.6501	4.124	4.445	17.414	−8.100	29.958
Overall	15.300	2.2769	6.719	10.496	20.103	−5.630	36.229

^a^ Truncated Knapp–Hartung method was used for SE adjustment. ^b^ Based on t-distribution.

**Table 8:** Cisplatin IC_50_ in 48 and 72 h HepG2 cell cultures with subgroups and overall effect size- forest plot. Test of homogeneity.

	Chi-Square (Q Statistic)	df	Sig.
48 h	4536.587	10	<0.0005
72 h	8323.964	6	<0.0005
Overall	20,761.129	17	<0.0005

df—degrees of freedom, Sig.—significance.

**Table 9:** Cisplatin IC_50_ in 48 and 72 h HepG2 cell cultures with subgroups and overall effect size- forest plot. Test of subgroup homogeneity.

	Chi-Square (Q Statistic)	df	Sig.
Time	3.054	1	0.081

df—degrees of freedom, Sig.—significance.

**Table 10:** Cisplatin IC_50_ in 48 and 72 h HepG2 cell cultures with subgroups and overall effect size- forest plot. Heterogeneity measures.

Time	Measure	Value
48 h	Tau-squared	106.532
	H-squared	1095.319
	I-squared (%)	99.9
72 h	Tau-squared	47.775
	H-squared	784.171
	I-squared (%)	99.9
Overall	Tau-squared	92.291
	H-squared	1947.583
	I-squared (%)	99.9

**Table 11:** Cisplatin IC_50_ in 48 and 72 h HepG2 cell cultures. Egger’s regression-based test ^a^.

	Parameter	Coefficient	Std. Error	t	Sig. (2-Tailed)	95% Confidence Interval	
						Lower	Upper
48 h	(Intercept)	12.555	4.9520	2.535	0.032	1.353	23.757
	SE ^c^	7.174	5.1441	1.395	0.197	−4.463	18.811
	Time = 48 h	0 ^b^	-	-	-	-	-
72 h	(Intercept)	6.470	3.0775	2.102	0.089	−1.441	14.380
	SE ^c^	8.091	4.0300	2.008	0.101	−2.268	18.450
	Time = 72 h	0 ^b^	-	-	-	-	-
Overall	(Intercept)	6.786	3.6942	1.837	0.086	−1.088	14.660
	SE ^c^	7.527	3.4813	2.162	0.047	0.107	14.947
	Time = 48 h	5.501	4.0986	1.342	0.199	−3.235	14.237
	Time = 72 h	0 ^b^	-	-	-	-	-

^a^ Random effects meta-regression with the truncated Knapp–Hartung SE adjustment. ^b^ This parameter was set to zero because it is redundant. ^c^ Standard error of effect size.

**Table 12:** IC_50_ at 48 and 72 h MCF-7 cell cultures with subgroups and overall effect size forest plot. Effect size estimates for individual studies.

	ID	Study	Effect Size	Std. Error ^a^	t	95% Confidence Interval		Weight	Weight (%)	Ref.
						Lower	Upper			
48 h	1	Chen et al., 2016	31.200	1.0392	30.022	29.163	33.237	0.019	4.2	[23]
	2	Ma et al., 2018	13.310	1.6743	7.950	10.028	16.592	0.018	4.0	[24]
	3	Mo et al., 2018	21.250	1.8879	11.256	17.550	24.950	0.018	4.0	[54]
	4	Purushothaman 2018	5.100	0.1328	38.406	4.840	5.360	0.019	4.3	[55]
	5	Song et al., 2018	11.200	1.2702	8.818	8.711	13.689	0.019	4.1	[42]
	6	Wei et al., 2018	10.050	0.4042	24.866	9.258	10.842	0.019	4.3	[43]
	7	Yilmaz et al., 2018a	24.000	2.3094	10.392	19.474	28.526	0.018	3.9	[56]
	8	Yilmaz et al., 2018b	11.680	0.4215	27.713	10.854	12.506	0.019	4.3	[57]
	9	Fei et al., 2019	6.300	0.2425	25.981	5.825	6.775	0.019	4.3	[28]
	10	Song et al., 2019	13.310	1.6743	7.950	10.028	16.592	0.018	4.0	[30]
	11	Chen J. et al., 2020	21.800	1.2702	17.163	19.311	24.289	0.019	4.1	[31]
	12	Golbarghi et al., 2020	20.100	2.0207	9.947	16.139	24.061	0.018	4.0	[58]
	13	Icsel et al., 2020	10.570	0.2252	46.943	10.129	11.011	0.019	4.3	[59]
	14	Li et al., 2020	15.600	0.3266	47.765	14.960	16.240	0.019	4.3	[33]
72 h	15	Popłoński et al., 2018	8.270	0.6062	13.642	7.082	9.458	0.019	4.2	[60]
	16	Praharova et al., 2018	16.600	0.2021	82.149	16.204	16.996	0.019	4.3	[61]
	17	Kostrhunov et al., 2019	13.000	1.7321	7.506	9.605	16.395	0.018	4.0	[62]
	18	Subarkhan et al., 2019	4.240	0.1617	26.228	3.923	4.557	0.019	4.3	[63]
	19	Abo-Ghalia et al., 2020	8.897	0.2136	41.649	8.478	9.316	0.019	4.3	[50]
	20	Krzywik et al., 2020a	5.812	0.8702	6.679	4.107	7.518	0.019	4.2	[64]
	21	Krzywik et al., 2020b	7.140	0.4062	17.576	6.344	7.936	0.019	4.3	[65]
	22	Lazaro et al., 2020	25.600	0.2858	89.581	25.040	26.160	0.019	4.3	[66]
	23	Mastalarz et al., 2020	12.600	1.5011	8.394	9.658	15.542	0.019	4.1	[67]
	24	Zang et al., 2022	5.020	0.2164	23.201	4.596	5.444	0.019	4.3	[52]

^a^ Truncated Knapp-Hartung method is used for SE adjustment.

**Table 13:** Cisplatin IC_50_ in 48 and 72 h MCF-7 cell cultures. Effect size estimates for subgroup analysis.

	Effect Size	Std. Error ^a^	t	95% Confidence Interval		95% Prediction Interval ^b^	
				Lower	Upper	Lower	Upper
48 h	15.271	1.9724	7.742	11.010	19.532	−1.140	31.682
72 h	10.703	2.0926	5.115	5.969	15.437	−5.192	26.599
Overall	13.347	1.4837	8.996	10.278	16.416	−1.867	28.560

^a^ Truncated Knapp–Hartung method was used for SE adjustment. ^b^ Based on t-distribution.

**Table 14:** Cisplatin IC_50_ in 48 and 72 h MCF-7 cell cultures. Test of homogeneity.

	Chi-Square (Q Statistic)	df	Sig.
48 h	2075.359	13	<0.0005
72 h	5955.162	9	<0.0005
Overall	8220.451	23	<0.0005

df—degrees of freedom, Sig.—significance.

**Table 15:** Cisplatin IC_50_ in 48 and 72 h MCF-7 cell cultures. Test of subgroup homogeneity.

	Chi-Square (Q Statistic)	df	Sig.
Time	2.523	1	0.112

df—degrees of freedom, Sig.—significance.

**Table 16:** Cisplatin IC_50_ in 48 and 72 h MCF-7 cell cultures. Heterogeneity measures.

Time	Measure	Value
48 h	Tau-squared	52.843
	H-squared	341.859
	I-squared (%)	99.7
72 h	Tau-squared	43.135
	H-squared	501.371
	I-squared (%)	99.8
Overall	Tau-squared	51.613
	H-squared	490.193
	I-squared (%)	99.8

**Table 17:** Cisplatin IC_50_ in 48 and 72 h MCF-7 cell cultures. Egger’s regression-based test ^a^.

	Parameter	Coefficient	Std. Error	t	Sig. (2-Tailed)	95% Confidence Interval	
						Lower	Upper
48 h	(Intercept)	9.150	2.8319	3.231	0.007	2.980	15.320
	SE ^b^	5.878	2.2378	2.627	0.022	1.003	10.754
72 h	(Intercept)	10.245	3.3436	3.064	0.015	2.535	17.955
	SE ^b^	0.755	4.1216	0.183	0.859	−8.749	10.260
Overall	(Intercept)	9.036	2.1195	4.263	<0.001	4.640	13.432
	SE ^b^	5.015	1.9291	2.600	0.016	1.014	9.016

^a^ Random effects meta-regression with the Truncated Knapp–Hartung SE adjustment. ^b^ Standard error of effect size.

## Data Availability

Data are contained within the article or Supplementary Material.

## Supplementary Materials

The following supporting information can be downloaded at: https://www.mdpi.com/article/10.3390/molecules28155761/s1. File S1: Table SA1. Source data for HeLa cell lines used in the analysis. Table SA2. Cisplatin IC50 deviations from average in 48 h HeLa cell cultures. Effects for individual studies. Table SA3. Cisplatin IC50 deviation from average in 48 h HeLa cell cultures. Effects for subgroup analysis Figure SA1. Cisplatin IC50 deviations from average in 48 h HeLa cell cultures—random forest plot. File S2: Table SB1. Source data for HepG2 cell lines included in the analysis. Table SB2. Cisplatin IC50 deviation from respective average in 48 h and 72 h HepG2 cell cultures. Effects for individual studies. Table SB3. Cisplatin IC50 deviations from respective average in 48 h and 72 h HepG2 cell cultures. Effects for subgroup analysis. Figure SB1. Cisplatin IC50 deviations from respective average in 48 h and 72 h HepG2 cell cultures—random forest. File S3: Table SC1. Source data for MCF-7 cell lines included in the analysis. Table SC2. Cisplatin IC50 deviation from respective average in 48 h and 72 h MCF7 cell cultures. Effects for individual studies. Table SC3. Cisplatin IC50 deviation from respective average in 48 h and 72 h MCF7 cell cultures. Effects for subgroup analysis. Figure SC1. Cisplatin IC50 deviation from respective average in 48 h and 72 h MCF7 cell cultures—random forest plot.
