# Supplementary material for: Disparities in Cisplatin-Induced Cytotoxicity—A Meta-Analysis of Selected Cancer Cell Lines
Source: Molecules. 2023 Jul 30;28(15):5761. doi: 10.3390/molecules28155761 (PMC10421281; doi:10.3390/molecules28155761)

Article

Disparities in Cisplatin-Induced Cytotoxicity—A Meta-Analysis of Selected Cancer Cell Lines

Małgorzata Ćwiklińska-Jurkowska [1], Małgorzata Wiese-Szadkowska [2,*], Sabina Janciauskiene [3] and Renata Paprocka [4,*]

[1] Department of Biostatistics and Biomedical Systems Theory, Faculty of Pharmacy, Ludwik Rydygier Collegium Medicum, Nicolaus Copernicus University in Toruń, Jagiellońska Str. 15, 87-067 Bydgoszcz, Poland; mjurkowska@cm.umk.pl
[2] Department of Immunology, Faculty of Pharmacy, Ludwik Rydygier Collegium Medicum, Nicolaus Copernicus University in Toruń, M. Curie-Sklodowska Str. 9, 85-094 Bydgoszcz, Poland
[3] Department of Respiratory Medicine, Biomedical Research in Endstage and Obstructive Lung Disease Hannover (BREATH), German Center for Lung Research (DZL), Hannover Medical School, 30625 Hannover, Germany; janciauskiene.sabina@mh-hannover.de
[4] Department of Organic Chemistry, Faculty of Pharmacy, Ludwik Rydygier Collegium Medicum, Nicolaus Copernicus University in Toruń, Jurasza Str. 2, 85-089 Bydgoszcz, Poland
* Correspondence: mwiese@cm.umk.pl (M.W.-S.); renata.bursa@cm.umk.pl (R.P.)

Table SA1. Source data for HeLa cell lines used in the analysis.

ID	Study name	Time	Method	Culture density (cells/well)	Mean of Cisplatin IC_{50}	SD	Number of replicates n	Ref.
1	Chen et al., 2016	48 h	MTT	4×10^3	15,3	1,9	3	[23]
2	Ma et al., 2018	48 h	MTT	ND	9,82	0,52	3	[24]
3	Qi et al., 2018	48 h	MTT	10^4	15,42	3,7	3	[25]
4	Zhang et al., 2018	48 h	MTT	8×10^3	7,1	1,2	3	[26]
5	Reddy et al., 2018	48 h	MTT	ND	3,25	0,28	3	[27]
6	Fei et al., 2019	48 h	MTT	$3\text{-}5 \times 10^3$	13,05	3,04	3	[28]
7	Song et al., 2019	48 h	MTT	3×10^3	9,82	0,52	3	[29]
8	Khan et al., 2019	48 h	MTT	4×10^3	7,58	0,82	5	[30]
9	Chen J. et al., 2020	48 h	MTT	4×10^3	15	2	3	[31]
10	Chen C. et al., 2020	48 h	MTT	ND	10,3	1,13	4	[32]
11	Liang et al., 2020	48 h	MTT	ND	9,45	0,25	3	[33]
12	Li et al., 2020	48 h	MTT	4×10^3	12,9	0,6	6	[34]
13	Pérez-Villanueva et al., 2021	48 h	MTT	7×10^3	18,5	5,20	3	[35]
14	Zeng et al., 2021	48 h	CCK-8 kit*	5×10^3	37,37	2,01	3	[36]
	Overall average (48 h)				*13,20*	*8,03*		

Tab. SA2. Cisplatin IC_{50} deviations from average in 48 h HeLa cell cultures. Effects for individual studies.

	ID	Study	Effect Size	Std. Error[a]	t	Sig. (2-tailed)	95% Confidence Interval Lower	Upper	Weight	Weight (%)
48 h	1	Chen et al., 2016	2,096	1,0970	1,910	,056	-,054	4,246	,015	7,2
	2	Ma et al., 2018	-3,384	,3002	-11,273	,000	-3,973	-2,796	,016	7,3
	3	Qi et al., 2018	2,216	2,1362	1,037	,300	-1,971	6,403	,015	6,8
	4	Reddy et al., 2018	-9,954	,1617	-61,576	,000	-10,271	-9,637	,016	7,3
	5	Zhang et al., 2018	-6,104	,6928	-8,811	,000	-7,462	-4,746	,016	7,3
	6	Fei et al., 2019	-,154	1,7551	-,088	,930	-3,594	3,286	,015	7,0
	7	Khan et al., 2019	-5,624	,3667	-15,337	,000	-6,343	-4,906	,016	7,3
	8	Song et al., 2019	-3,384	,3002	-11,273	,000	-3,973	-2,796	,016	7,3
	9	Chen J. et al., 2020	1,796	1,1547	1,555	,120	-,467	4,059	,015	7,2
	10	Chen C. et al., 2020	-2,904	,5650	-5,140	<,001	-4,012	-1,797	,016	7,3
	11	Li et al., 2020	-,304	,2449	-1,242	,214	-,784	,176	,016	7,3
	12	Liang et al., 2020	-3,754	,1443	-26,010	,000	-4,037	-3,471	,016	7,3
	13	Pérez-Villanueva et al., 2021	5,296	3,0000	1,765	,078	-,584	11,176	,014	6,4
	14	Zeng et al., 2021	24,166	1,1605	20,824	,000	21,891	26,440	,015	7,2

[a]Truncated Knapp-Hartung method is used for SE adjustment.

Tab. SA3. Cisplatin IC_{50} deviation from average in 48 h HeLa cell cultures. Effects for subgroup analysis.

	Effect Size	Std. Error[a]	t	Sig. (2-tailed)	95% Confidence Interval Lower	Upper	95% Prediction Interval[b] Lower	Upper
48 h	-,094	2,1523	-,044	,966	-4,744	4,555	-18,064	17,875

[a]Truncated Knapp-Hartung method is used for SE adjustment. [b]Based on t-distribution.

Fig. S1A. Cisplatin IC_{50} deviations from average in 48 h HeLa cell cultures - random forest plot.

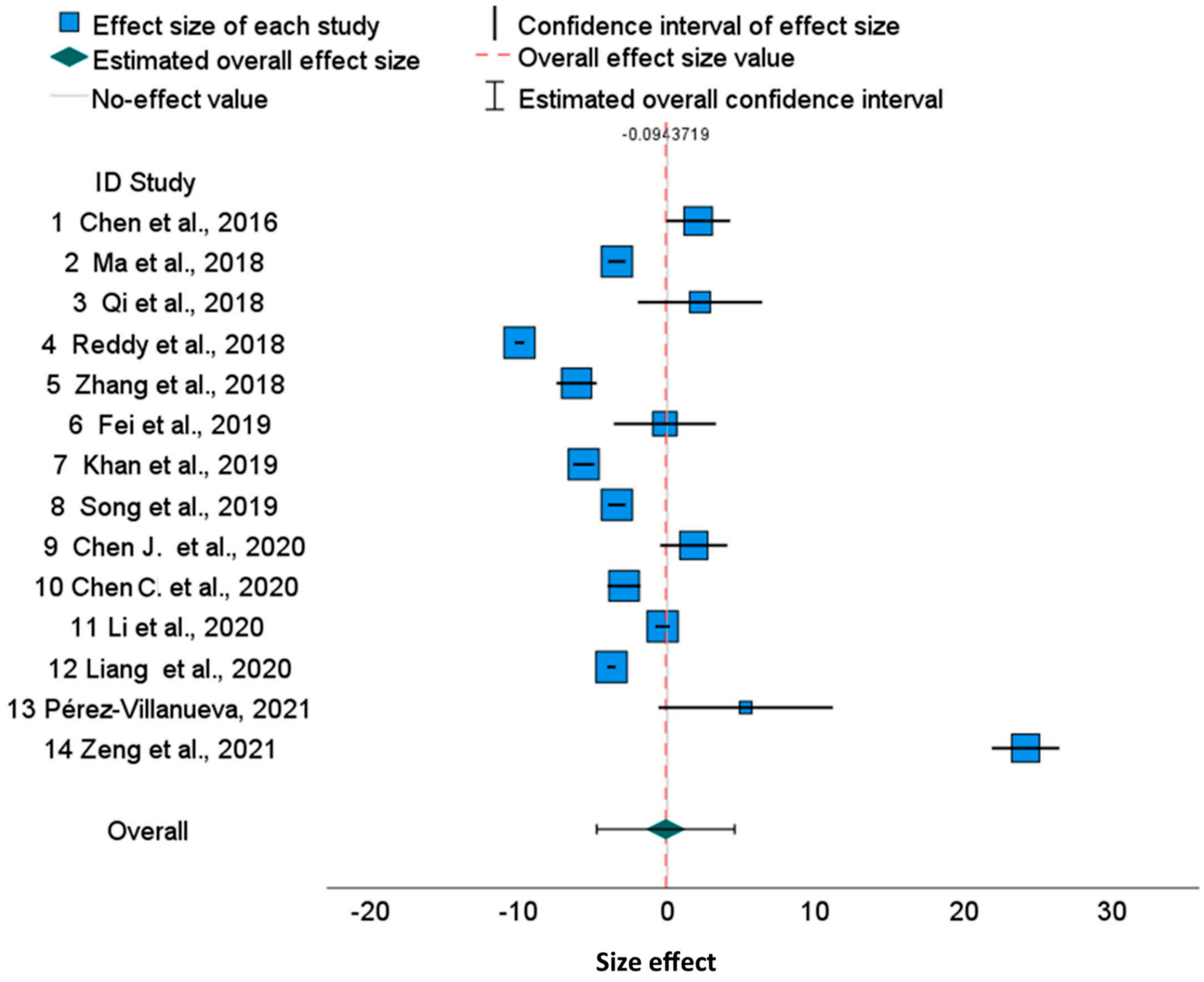

Supplement: Supplementary file 1 [file molecules-28-05761-s001.zip › Appendix A HeLa 20.07.2023.pdf]
