# Supplementary material for: Disparities in Cisplatin-Induced Cytotoxicity—A Meta-Analysis of Selected Cancer Cell Lines
Source: Molecules. 2023 Jul 30;28(15):5761. doi: 10.3390/molecules28155761 (PMC10421281; doi:10.3390/molecules28155761)

Article

Disparities in Cisplatin-Induced Cytotoxicity—A Meta-Analysis of Selected Cancer Cell Lines

Małgorzata Ćwiklińska-Jurkowska [1], Małgorzata Wiese-Szadkowska [2,*], Sabina Janciauskiene [3] and Renata Paprocka [4,*]

1 Department of Biostatistics and Biomedical Systems Theory, Faculty of Pharmacy, Ludwik Rydygier Collegium Medicum, Nicolaus Copernicus University in Toruń, Jagiellońska Str. 15, 87-067 Bydgoszcz, Poland; mjurkowska@cm.umk.pl
2 Department of Immunology, Faculty of Pharmacy, Ludwik Rydygier Collegium Medicum, Nicolaus Copernicus University in Toruń, M. Curie-Sklodowska Str. 9, 85-094 Bydgoszcz, Poland
3 Department of Respiratory Medicine, Biomedical Research in Endstage and Obstructive Lung Disease Hannover (BREATH), German Center for Lung Research (DZL), Hannover Medical School, 30625 Hannover, Germany; janciauskiene.sabina@mh-hannover.de
4 Department of Organic Chemistry, Faculty of Pharmacy, Ludwik Rydygier Collegium Medicum, Nicolaus Copernicus University in Toruń, Jurasza Str. 2, 85-089 Bydgoszcz, Poland
* Correspondence: mwiese@cm.umk.pl (M.W.-S.); renata.bursa@cm.umk.pl (R.P.)

Table SB1. Source data for HepG2 cell lines included in the analysis.

ID	Study name	Time	Method	Culture density [cells/well]	Mean of Cisplatin IC_{50}	SD	Number of replicates n	Ref.
1	Chen et al., 2016	48 h	MTT	4×10^3	25,8	3,9	3	[23]
2	Deng et al., 2018	48h	MTT	ND	17,23	0,24	3	[40]
3	Qin et al., 2018	48 h	MTT	5.0 × 10^3	15,16	1,21	5	[41]
4	Song et al., 2018	48 h	MTT	5 x 10^4	8,2	0,17	3	[42]
5	Wei et al., 2018	48 h	MTT	5.0 × 10^3	16,78	1,02	6	[43]
6	Zhang et al., 2018	48 h	MTT	8 x 10^3	24,7	2,2	3	[27]
7	Chai et al., 2019	48 h	MTT	10,000	12,5	1,8	3	[44]
8	Fei et al., 2019	48 h	MTT	3-5× 10^3	7,63	1,6	3	[28]
9	Qin et al., 2019	48 h	MTT	5.0 × 10^3	17,13	1,05	5	[45]
10	Liang et al., 2020	48 h	MTT	ND	10,28	0,77	3	[34]
11	Zeng et al., 2021	48 h	CCK-8 kit	5 × 10^3	43,81	1,62	3	[36]
					18.11	10.37		
12	Huang et al., 2018	72 h	MTT	5 x 10^4	5,03	0,64	3	[46]
13	Li et al., 2018	72 h	MTT	5.0 x 10^4	8,36	0,92	3	[47]
14	Hua et al., 2019	72 h	MTT	5.0 x 10^4	9,33	0,72	3	[48]
15	Li et al., 2019	72 h	MTT	5 x 10^3	18,6	0,3	3	[49]
16	Abo-Ghalia, 2020	72 h	MTT	ND	10,93	0,962	3	[50]
17	Tong et al., 2020	72 h	MTT	4–6 x 10^3	22,3	3,2	3	[51]
18	Zang et al., 2022	72 h	MTT	ND	2,68	0,12	6	[52]
					11.03	7.07		Summary for 72 h
					15.36	9.67		Overall summary

Tab. SB2. Cisplatin IC_{50} deviation from respective average in 48 h and 72 h HepG2 cell cultures. Effects for individual studies.

	ID	Study	Effect Size	Std. Error[a]	t	Sig. (2-tailed)	95% Confidence Interval Lower	Upper	Weight	Weight (%)
48 h	1	Chen et al., 2016	7,689	2,2517	3,415	<,001	3,276	12,102	,012	5,3
	2	Deng et al., 2018	-,881	,1386	-6,357	<,001	-1,152	-,609	,013	5,6
	3	Qin et al., 2018	-2,951	,5411	-5,453	<,001	-4,012	-1,890	,013	5,6
	4	Song et al., 2018	-9,911	,0981	-100,978	,000	-10,103	-9,719	,013	5,6
	5	Wei et al., 2018	-1,331	,4164	-3,196	,001	-2,147	-,515	,013	5,6
	6	Zhang et al., 2018	6,589	1,2702	5,188	<,001	4,100	9,079	,012	5,5
	7	Chai et al., 2019	-5,611	1,0392	-5,399	<,001	-7,648	-3,574	,012	5,5
	8	Fei et al., 2019	-10,481	,9238	-11,346	,000	-12,291	-8,670	,012	5,6
	9	Qin et al., 2019	-,981	,4696	-2,089	,037	-1,901	-,061	,013	5,6
	10	Liang et al., 2020	-7,831	,4446	-17,615	,000	-8,702	-6,960	,013	5,6
	11	Zeng et al., 2021	25,699	,9353	27,477	,000	23,866	27,532	,012	5,6
72 h	12	Huang et al., 2018	-6,003	,3695	-16,246	,000	-6,727	-5,279	,013	5,6
	13	Li et al., 2018	-2,673	,5312	-5,032	<,001	-3,714	-1,632	,013	5,6
	14	Hua et al., 2019	-1,703	,4157	-4,096	<,001	-2,518	-,888	,013	5,6
	15	Li et al., 2019	7,567	,1732	43,689	,000	7,228	7,907	,013	5,6
	16	Abo-Ghalia et al., 2020	-,103	,5554	-,185	,853	-1,191	,986	,013	5,6
	17	Tong et al., 2020	11,267	1,8475	6,099	<,001	7,646	14,888	,012	5,4
	18	Zang et al., 2022	-8,353	,0490	-170,502	,000	-8,449	-8,257	,013	5,6

[a]Truncated Knapp-Hartung method is used for SE adjustment.

Tab. SB3. Cisplatin IC_{50} deviations from respective average in 48 h and 72 h HepG2 cell cultures. Effects for subgroup analysis.

					95% Confidence Interval		95% Prediction Interval[b]	
	Effect Size	Std. Error[a]	t	Sig. (2-tailed)	Lower	Upper	Lower	Upper
48 h	-,045	3,1266	-,014	,989	-7,011	6,922	-24,441	24,352
72 h	-,104	2,6501	-,039	,970	-6,588	6,381	-19,133	18,925
Overall	-,061	2,1167	-,029	,977	-4,527	4,405	-19,495	19,373

[a]Truncated Knapp-Hartung method is used for SE adjustment. [b]Based on t-distribution.

Fig. SB1. Cisplatin IC_{50} deviations from respective average in 48 h and 72 h HepG2 cell cultures-random forest.

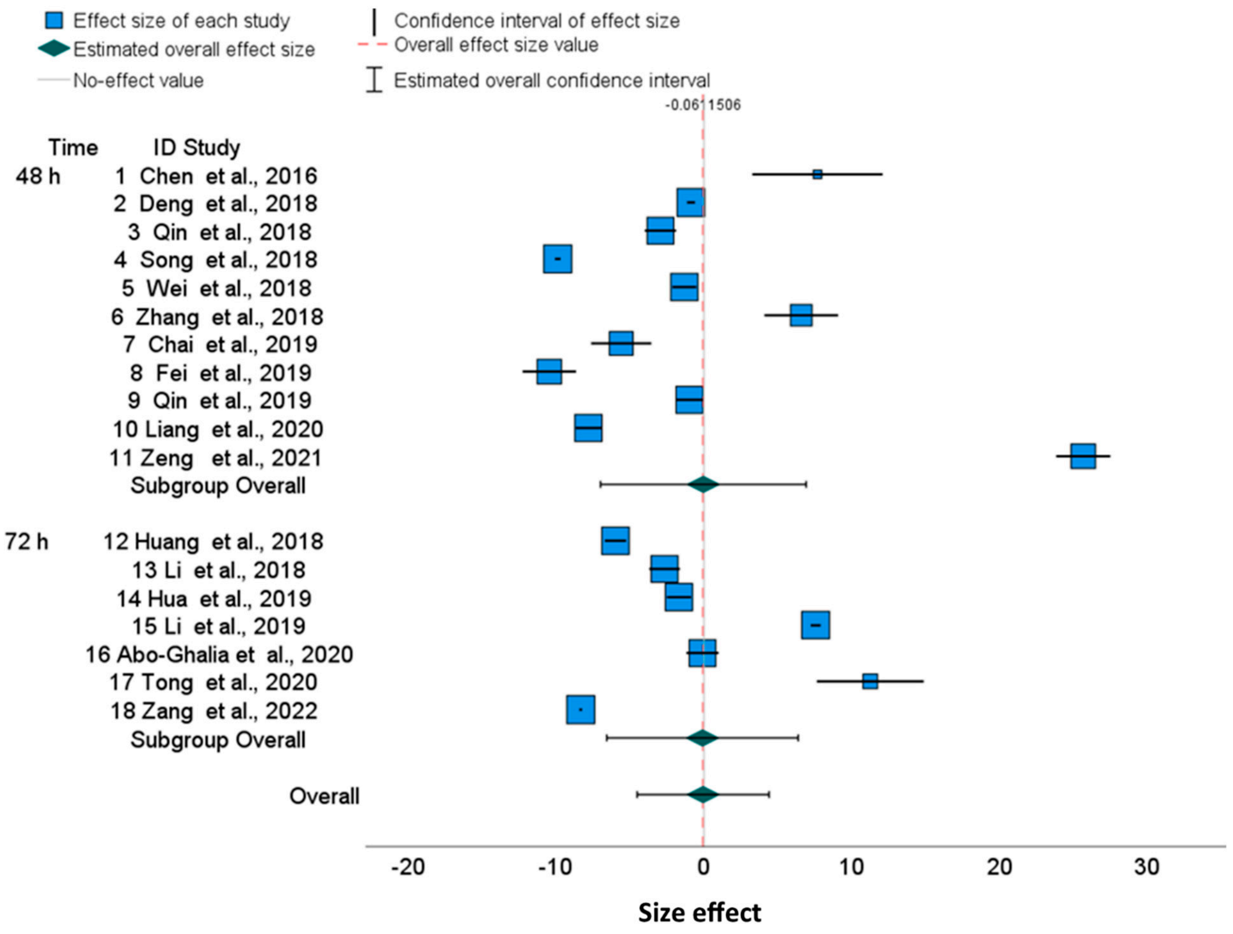

Supplement: Supplementary file 1 [file molecules-28-05761-s001.zip › Appendix B HEPG2 20.07.2023.pdf]
