# Supplementary material for: Disparities in Cisplatin-Induced Cytotoxicity—A Meta-Analysis of Selected Cancer Cell Lines
Source: Molecules. 2023 Jul 30;28(15):5761. doi: 10.3390/molecules28155761 (PMC10421281; doi:10.3390/molecules28155761)

Article

Disparities in Cisplatin-Induced Cytotoxicity—A Meta-Analysis of Selected Cancer Cell Lines

Małgorzata Ćwiklińska-Jurkowska [1], Małgorzata Wiese-Szadkowska [2,*], Sabina Janciauskiene [3] and Renata Paprocka [4,*]

1 Department of Biostatistics and Biomedical Systems Theory, Faculty of Pharmacy, Ludwik Rydygier Collegium Medicum, Nicolaus Copernicus University in Toruń, Jagiellońska Str. 15, 87-067 Bydgoszcz, Poland; mjurkowska@cm.umk.pl
2 Department of Immunology, Faculty of Pharmacy, Ludwik Rydygier Collegium Medicum, Nicolaus Copernicus University in Toruń, M. Curie-Sklodowska Str. 9, 85-094 Bydgoszcz, Poland
3 Department of Respiratory Medicine, Biomedical Research in Endstage and Obstructive Lung Disease Hannover (BREATH), German Center for Lung Research (DZL), Hannover Medical School, 30625 Hannover, Germany; janciauskiene.sabina@mh-hannover.de
4 Department of Organic Chemistry, Faculty of Pharmacy, Ludwik Rydygier Collegium Medicum, Nicolaus Copernicus University in Toruń, Jurasza Str. 2, 85-089 Bydgoszcz, Poland
* Correspondence: mwiese@cm.umk.pl (M.W.-S.); renata.bursa@cm.umk.pl (R.P.)

Table SC1. Source data for MCF-7 cell lines included in the analysis.

ID	Study name	Time	Method	Culture density [cells/well]	Cisplatin IC_{50}	SD	Number of replicates n	Ref.
1	Chen et al., 2016	48 h	MTT	4×10^3	31,2	1,8	3	[23]
2	Ma et al., 2018	48 h	MTT	ND	13,31	2,9	3	[24]
3	Mo et al., 2018	48 h	MTT	5×10^4	21,25	3,27	3	[54]
4	Purushothaman 2018	48 h	SRB	1×10^6	5,1	0,23	3	[55]
5	Song et al., 2018	48 h	MTT	2×10^3	11,2	2,2	3	[42]
6	Wei et al., 2018	48 h	MTT	5.0×10^3	10,05	0,99	6	[43]
7	Yilmaz et al., 2018 a	48 h	ATP	5×10^3	24	4	3	[56]
8	Yilmaz et al., 2018 b	48 h	ATP	5×10^3	11,68	0,73	3	[57]
9	Fei et al., 2019	48 h	MTT	$3\text{-}5 \times 10^3$	6,3	0,42	3	[28]
10	Song et al., 2019	48 h	MTT	3×10^3	13,31	2,9	3	[30]
11	Chen J. et al., 2020	48 h	MTT	4×10^3	21,8	2,2	3	[31]
12	Golbarghi et al., 2020	48 h	SRB	10^4	20,1	3,5	3	[58]
13	Icsel et al., 2020	48 h	ATP	1×10^3	10,57	0,39	3	[59]
14	Li et al., 2020	48 h	MTT	4×10^3	15,6	0,8	6	[33]
	Average for 48 h				***15,39***	***7,35***		
15	Popłoński et al., 2018	72 h	SRB	10^4	8,27	2,1	12	[60]
16	Praharova et al., 2018	72 h	MTT	5×10^3	16,6	0,7	12	[61]
17	Kostrhunov et al., 2019	72 h	MTT	8×10^3	13	3	3	[62]
18	Subarkhan et al., 2019	72 h	MTT	ND	4,24	0,28	3	[63]
19	Abo-Ghalia et al., 2020	72 h	MTT	ND	8,897	0,37	3	[50]
20	Krzywik et al., 2020 a	72 h	SRB	0.75×10^4	5,8122	2,610 6	9	[64]
21	Krzywik et al., 2020 b	72 h	SRB	0.75×10^4	7,1398	1,218 7	9	[65]
22	Lazaro et al., 2020	72 h	MTT	ND	25,6	0,7	6	[66]
23	Mastalarz et al., 2020	72 h	SRB	10^4	12,6	2,6	3	[67]
24	Zang et al., 2022	72 h	MTT	ND	5,02	0,53	6	[52]
	Average for 72 h				***10,72***	***6,26***		
	Overall average				***13,44***	***7,27***		

Tab. SC2. Cisplatin IC_{50} deviation from respective average in 48 h and 72 h MCF7 cell cultures. Effects for individual studies.

	ID	Study	Effect Size	Std. Error[a]	t	Sig. (2-tailed)	95% Confidence Interval		Weight	Weight (%)
							Lower	Upper		
48 h	1	Chen et al., 2016	15,809	1,0392	15,212	,000	13,772	17,846	,019	4,2
	2	Ma et al., 2018	-2,081	1,6743	-1,243	,214	-5,362	1,201	,019	4,0
	3	Mo et al., 2018	5,859	1,8879	3,104	,002	2,159	9,560	,019	4,0
	4	Purushothaman 2018	-10,291	,1328	-77,496	,000	-10,551	-10,030	,020	4,3
	5	Song et al., 2018	-4,191	1,2702	-3,299	<,001	-6,680	-1,701	,019	4,1
	6	Wei et al., 2018	-5,341	,4042	-13,214	,000	-6,133	-4,549	,020	4,3
	7	Yilmaz et al., 2018 a	8,609	2,3094	3,728	<,001	4,083	13,136	,018	3,9
	8	Yilmaz et al., 2018 b	-3,711	,4215	-8,804	,000	-4,537	-2,885	,020	4,3
	9	Fei et al., 2019	-9,091	,2425	-37,489	,000	-9,566	-8,615	,020	4,3
	10	Song et al., 2019	-2,081	1,6743	-1,243	,214	-5,362	1,201	,019	4,0
	11	Chen J. et al., 2020	6,409	1,2702	5,046	<,001	3,920	8,899	,019	4,1
	12	Golbarghi et al., 2020	4,709	2,0207	2,330	,020	,749	8,670	,018	3,9
	13	Icsel et al., 2020	-4,821	,2252	-21,410	,000	-5,262	-4,379	,020	4,3
	14	Li et al., 2020	,209	,3266	,641	,522	-,431	,849	,020	4,3
72 h	15	Popłoński et al., 2018	-2,448	,6062	-4,038	<,001	-3,636	-1,260	,020	4,2
	16	Praharova et al., 2018	1,209	,2021	5,984	<,001	,813	1,605	,020	4,3
	17	Kostrhunov et al., 2019	-2,391	1,7321	-1,380	,168	-5,785	1,004	,019	4,0
	18	Subarkhan et al., 2019	-11,151	,1617	-68,977	,000	-11,468	-10,834	,020	4,3
	19	Abo-Ghalia et al., 2020	-6,494	,2136	-30,398	,000	-6,912	-6,075	,020	4,3
	20	Krzywik et al., 2020 a	-9,579	,8702	-11,007	,000	-11,284	-7,873	,020	4,2
	21	Krzywik et al., 2020 b	-8,251	,4062	-20,311	,000	-9,047	-7,455	,020	4,3
	22	Lazaro et al., 2020	10,209	,2858	35,725	,000	9,649	10,769	,020	4,3
	23	Mastalarz et al., 2020	-2,791	1,5011	-1,859	,063	-5,733	,151	,019	4,1
	24	Zang et al., 2022	-10,371	,2164	-47,930	,000	-10,795	-9,947	,020	4,3

[a]Truncated Knapp-Hartung method is used for SE adjustment.

Tab. SC3. Cisplatin IC_{50} deviation from respective average in 48 h and 72 h MCF7 cell cultures. Effects for subgroup analysis.

	Effect Size	Std. Error[a]	t	Sig. (2-tailed)	95% Confidence Interval		95% Prediction Interval[b]	
					Lower	Upper	Lower	Upper
48 h	-,120	1,9724	-,061	,953	-4,381	4,142	-16,531	16,292
72 h	-4,217	2,0847	-2,023	,074	-8,933	,499	-20,052	11,617
Overall	-1,848	1,4674	-1,259	,221	-4,883	1,188	-16,891	13,195

[a]Truncated Knapp-Hartung method is used for SE adjustment. [b]Based on t-distribution.

Fig. SC1. Cisplatin IC_{50} deviation from respective average in 48 h and 72 h MCF7 cell cultures- random forest plot.

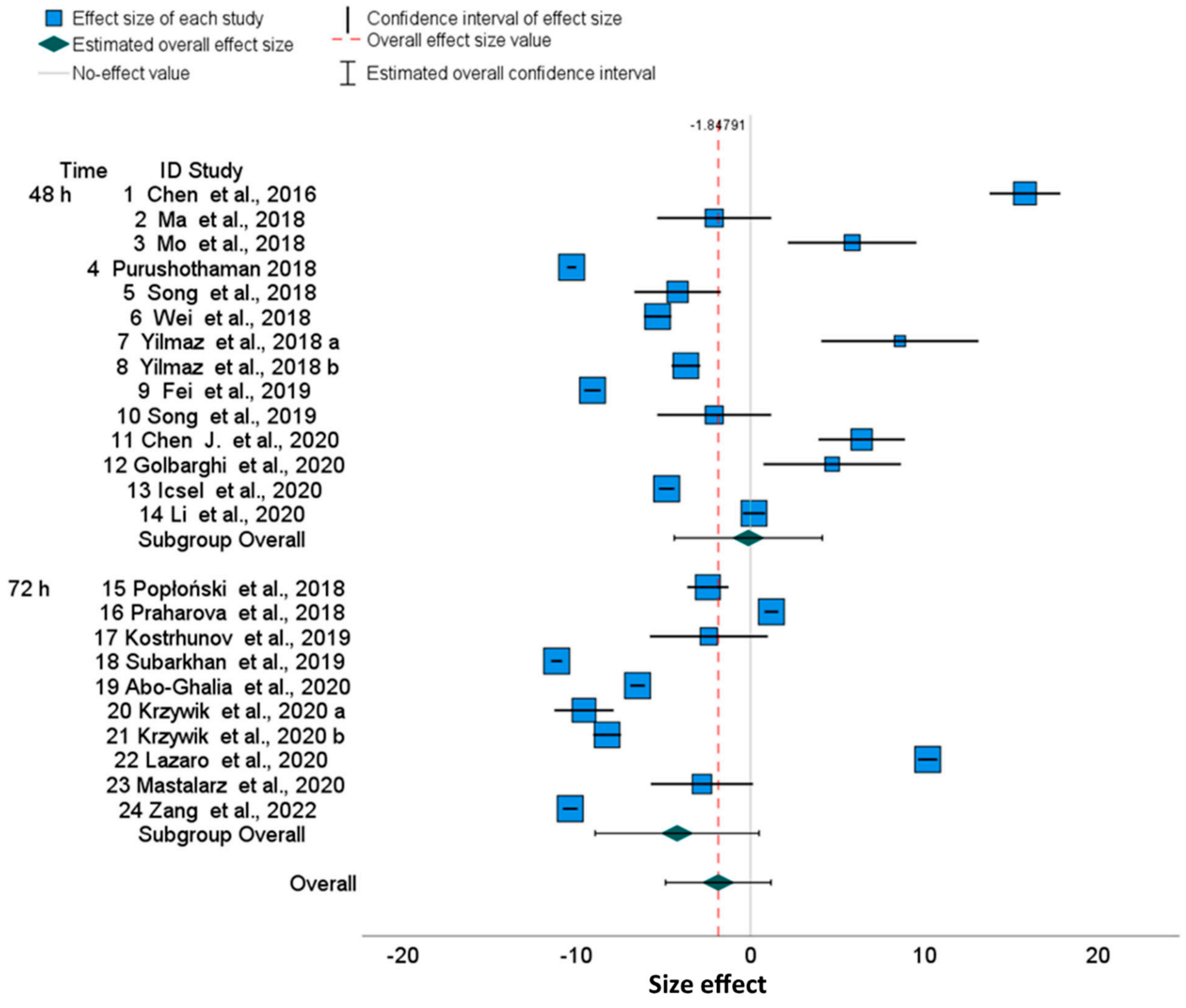

Supplement: Supplementary file 1 [file molecules-28-05761-s001.zip › Appendix C MCF-7 20.07.2023.pdf]
